# The SVZ stem cell niche–components, functions, and *in vitro* modelling

**DOI:** 10.3389/fcell.2023.1332901

**Published:** 2023-12-22

**Authors:** Nesil Eşiyok, Michael Heide

**Affiliations:** Research Group Brain Development and Evolution, German Primate Center, Leibniz Institute for Primate Research, Göttingen, Germany

**Keywords:** neocortex development, subventricular zone, basal progenitors, stem cell niche, ECM, vasculature

## Abstract

Neocortical development depends on the intrinsic ability of neural stem and progenitor cells to proliferate and differentiate to generate the different kinds of neurons in the adult brain. These progenitor cells can be distinguished into apical progenitors, which occupy a stem cell niche in the ventricular zone and basal progenitors, which occupy a stem cell niche in the subventricular zone (SVZ). During development, the stem cell niche provided in the subventricular zone enables the increased proliferation and self-renewal of basal progenitors, which likely underlie the expansion of the human neocortex. However, the components forming the SVZ stem cell niche in the developing neocortex have not yet been fully understood. In this review, we will discuss potential components of the SVZ stem cell niche, i.e., extracellular matrix composition and brain vasculature, and their possible key role in establishing and maintaining this niche during fetal neocortical development. We will also emphasize the potential role of basal progenitor morphology in maintaining their proliferative capacity within the stem cell niche of the SVZ. Finally, we will focus on the use of brain organoids to i) understand the unique features of basal progenitors, notably basal radial glia; ii) study components of the SVZ stem cell niche; and iii) provide future directions on how to improve brain organoids, notably the organoid SVZ, and make them more reliable models of human neocortical development and evolution studies.

## 1 Introduction

The neocortex is the most recently evolved brain structure and is widely considered as the seat of higher cognitive abilities (Rakic, 1995; Rakic, 2009; Borrell and Reillo, 2012). The size and the degree of folding of the neocortex exhibit strong diversity across different species culminating in the large and highly folded human neocortex (Rakic, 2009; Lui et al., 2011; Borrell and Reillo, 2012; Florio and Huttner, 2014; Dehay et al., 2015). Cortical development is a complex and intricately regulated process that relies on the proliferation and differentiation ability of cortical stem and progenitor cells to form the layered structure of the cortical wall (Götz and Huttner, 2005). All neurons of the mammalian cortex are essentially derived from neuroepithelial cells (Taverna et al., 2014). At early embryogenesis, the neuroepithelial cells divide symmetrically to amplify their number leading to lateral expansion of the telencephalon (Rakic, 1995). Later at the onset of neurogenesis, neuroepithelial cells transform into a type of apical progenitor cell called apical radial glia (aRG) (Götz and Huttner, 2005; Yamashita, 2013). Apical progenitors, including aRG, are localized in the ventricular zone (VZ)–the primary germinal zone, which directly lines the ventricle. Apical radial glia show a highly polarized, elongated morphology with an apical process reaching to the ventricular surface and a basal process extending to the pial surface in the developing cortex (Kriegstein and Götz, 2003; Lui et al., 2011; Florio and Huttner, 2014). At the early stages of neurogenesis, aRG mainly divide symmetrically to give rise to more aRG; later they progressively switch to asymmetric divisions to self-renew and produce mainly basal progenitors (see below) or, to a lesser extent, neurons of the developing neocortex (Kriegstein and Götz, 2003; Florio and Huttner, 2014). Throughout the cell cycle aRG remain attached to the ventricular surface. While being attached, the cell nucleus moves within the VZ depending on the phase of the cell cycle in a specific process called interkinetic nuclear migration (INM) (Schenk et al., 2009; Taverna et al., 2014). During S-phase the nucleus migrates towards the basal side of the VZ and then it moves in apical direction to the ventricular surface, where mitosis takes place (Taverna et al., 2014). This movement is necessary for positioning the nucleus close to the centrosome, which is apically localized due to the presence of the primary cilium (Wilsch-Bräuninger and Huttner, 2021; Zaidi et al., 2022). Thus, mitosis of aRG is restricted to the ventricular surface. This means that the number of aRG mitoses is limited by the size of the ventricular surface. Accordingly, brain size would be limited by the available space for mitotic aRG at the ventricular surface. To overcome this spatial limitation, a second germinal zone basal to the ventricular zone (VZ), the so-called subventricular zone (SVZ), has emerged. The SVZ contains proliferative cells that are no longer attached to the ventricular surface and can divide anywhere within the SVZ (Smart et al., 2002). These cells are called basal progenitors (BPs) (Hansen et al., 2010; Fietz and Huttner, 2011; Lui et al., 2011; Reillo et al., 2011; Florio and Huttner, 2014).

**FIGURE 1:**
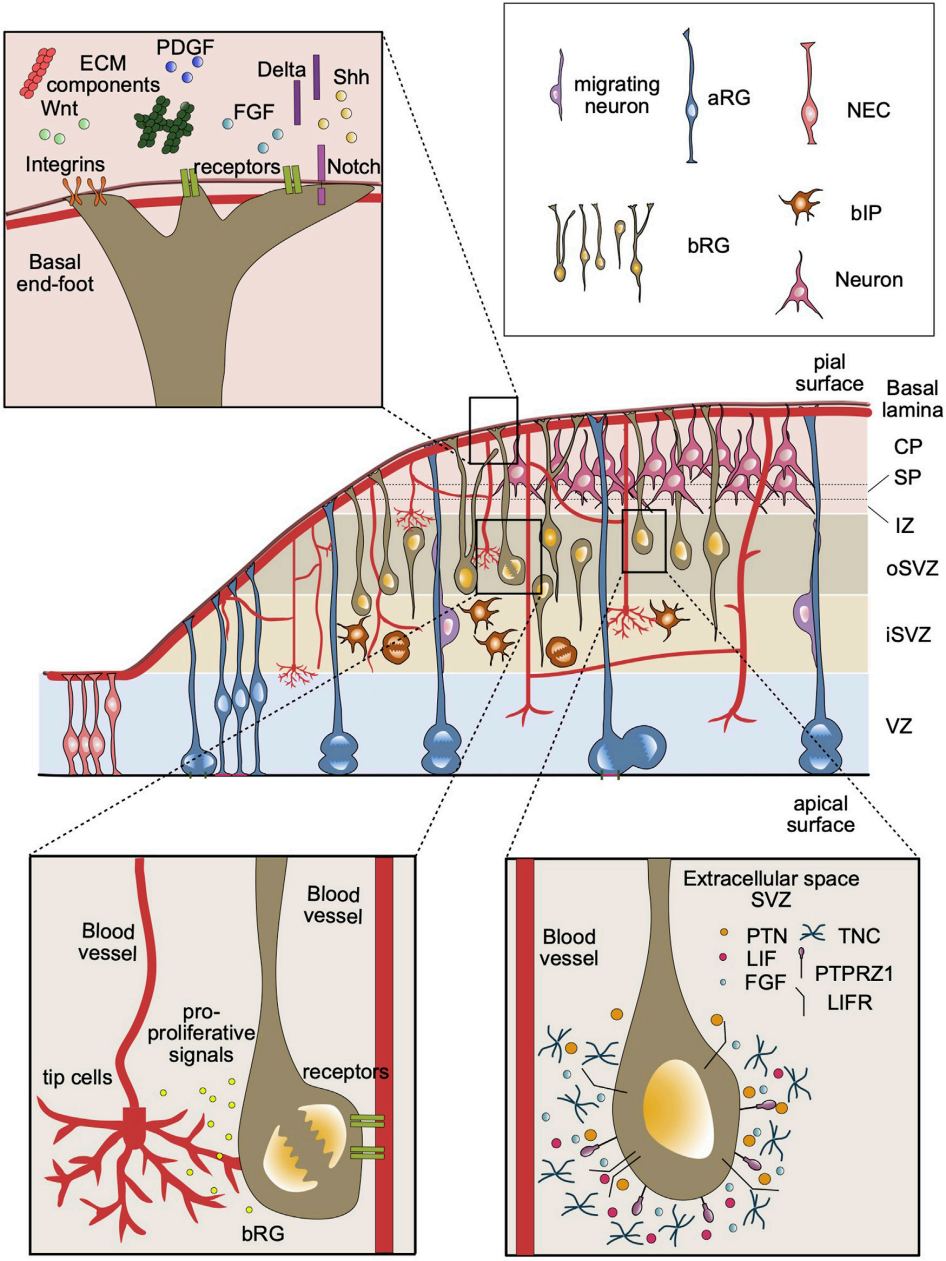
Overview of the components of SVZ stem cell niche in the developing neocortex. A developing cortical wall including all major cell types of a representative gyrencephalic mammal, such as human or ferret (middle panel). The ECM components and growth factors in the basal lamina can be detected by the cell surface receptors at the basal end-feet of bRG, including integrins and Notch (top left panel). Blood vessels secrete factors that may be received by basal progenitors in the SVZ. Basal progenitors can also establish contacts with basal lamina of the blood vessels, which may provide an additional source for signaling molecules (bottom left panel). The extracellular environment in the SVZ contains specific ECM proteins (e.g., TNC), receptor ligands (e.g., PTN, LIF) and growth factors (e.g., FGF). These components that are detected by bRG cell surface receptors, such as PTPRZ1 and LIFR, may activate intracellular signaling pathways to promote bRG proliferation (bottom right panel).

Basal progenitors are initially generated from oblique and/or horizontal asymmetrical divisions of aRG (Lamonica et al., 2013; Hansen et al., 2010; Shitamukai et al., 2011). As a consequence, the daughter cell destined to differentiate into a BP will lose contact to the ventricular surface and migrate out of the VZ–a process called delamination (Lamonica et al., 2013; Taverna et al., 2014). Along with the loss of the apical contact, mitosis of BPs is no longer restricted to the ventricular surface and can occur basically anywhere in the SVZ. In general, two types of BPs can be distinguished in the SVZ of the mammalian cortex based on their morphology and proliferative capacity: basal intermediate progenitors (bIPs) and basal radial glia (bRG, also called outer radial glia, oRG). bIPs are multipolar cells that can typically undergo one round of symmetrical neurogenic division to produce two neurons (Haubensak et al., 2004; Noctor et al., 2004). In rodents, the majority of bIPs are derived from the aRGs in the VZ, while in primates and gyrencephalic mammals, like ferrets, they are mostly generated within the SVZ from bRG and/or bIPs themselves (Haubensak et al., 2004; Miyata et al., 2004; Noctor et al., 2004). Moreover, unlike rodent bIPs, these bIPs can undergo a few rounds of proliferative divisions to expand their pool before undergoing neurogenic divisions (Fietz et al., 2010; Hansen et al., 2010). The other type of BP, the bRG, was initially defined by a basal process, which contacts the basal lamina, and by–in contrast to aRG–lacking an apical process, which contacts the ventricular surface (Fietz et al., 2010; Hansen et al., 2010; Reillo et al., 2011; LaMonica et al., 2012). In later studies, bRG with various morphologies were identified (Betizeau et al., 2013; Kalebic et al., 2019) (for details see below). In contrast to bIPs, bRG can undergo multiple rounds of symmetric proliferative divisions in the SVZ and expand their pool size significantly (Fietz et al., 2010; Shitamukai et al., 2011). Due to this high proliferative capacity, bRG are thought to be the key progenitor cell type underlying the evolutionary expansion of the mammalian neocortex (Borrell and Reillo, 2012; Florio and Huttner, 2014; Kalebic et al., 2019). Indeed, mammals with a large and folded neocortex, notably primates, exhibit a greater number of bRGs in the SVZ resulting in an expanded SVZ, which can be morphologically subdivided into an inner SVZ (iSVZ) and an outer SVZ (oSVZ) (Figure 1) (Smart et al., 2002; Dehay et al., 2015).

The high proliferative capacity of aRG and bRG strongly depends on the accessibility of oxygen, nutrients, growth factors and signaling molecules within their stem cell niche. Attached to the ventricular surface throughout the cell cycle, aRG have direct access to the cerebrospinal fluid (CSF) in the ventricle. The CSF contains critical signaling molecules required for the survival, proliferation, and neurogenic capacity of aRG including fibroblast growth factors (FGFs), insulin-like growth factors (IGFs), sonic hedgehog (Shh), retinoic acid, bone morphogenic proteins (BMPs), and Wnts (Johansson et al., 2008; Lehtinen and Walsh, 2011; Yeh et al., 2013). The access of aRG to these signaling molecules is largely mediated by the receptors on the apical membrane and notably on the primary cilium. For example, the glycoprotein megalin on the apical membrane binds to BMPs and Shhs in the CSF (McCarthy et al., 2002; Wicher et al., 2005). Upon ligand binding, the cytoplasmic domain of megalin is cleaved and translocated to the nucleus, where it likely influences the proliferation and cell fate decision of aRG by altering gene expression (McCarthy and Argraves, 2003). On the other hand, the striking ability of bRG to maintain a high proliferative capacity away from the ventricle–without access to the factors in the CSF–highlights that there must be other strategies to establish and maintain a stem cell niche for these cells within the SVZ.

In this review, we will describe potential components that define the stem cell niche in the SVZ during fetal neocortical development including specific ECM components and vasculature. We will then highlight the influence of this niche/microenvironment on the striking morphological heterogeneity and proliferation of bRG. Finally, we will discuss the current state of brain organoids as a model system to study bRG biology and provide potential future directions/strategies to improve bRG abundance in brain organoids by identifying the components of the stem cell niche in the SVZ.

## 2 The role of ECM in establishing the SVZ stem cell niche

Extracellular matrix (ECM) proteins are broadly expressed in the developing cortex and are associated with a multitude of processes ranging from regulation of neural stem cell and progenitor behavior (Pollen et al., 2015; Long and Huttner, 2019; Güven et al., 2020) to neuronal migration (Rakic, 1972; Sidman and Rakic, 1973) and axonal growth (Myers et al., 2011; Roumazeilles et al., 2018; Chui-Kuen Chan et al., 2020). The ECM is composed of various proteins including proteoglycans, laminins, dystroglycans and collagens (Barros et al., 2011; Long and Huttner, 2019; Ferent et al., 2020a), and constitutes around 40% of the developing cortex (Milošević et al., 2014). The interactions between ECM and neural cells play essential roles during cortical development and neurogenesis (Fietz et al., 2012; Long and Huttner, 2022). Transcriptome analyses of the human and mouse developing cortex revealed that increased expression of ECM components promotes proliferation and self-renewal of VZ and SVZ progenitors in the human neocortex (Fietz et al., 2012). Interestingly, in contrast to human bIPs, the expression of such ECM components was found to be downregulated in neurogenic mouse bIPs (Arai et al., 2011). In addition, a later study showed that the SOX9 transcription factor, expressed in human and ferret, but not in the mouse SVZ, promotes BP proliferation by regulating local ECM production. This highlights the role of ECM in the differential proliferative and self-renewal capacity of mouse vs. human basal progenitors (Güven et al., 2020). As a major ECM receptor, integrins seem to play an essential role in sustaining the proliferative capacity of basal progenitors (Fietz et al., 2010; Stenzel et al., 2014; Güven et al., 2020). Integrins, localized on the basal end-feet of the basal processes of bRG and aRG, transmit pro-proliferative signals from the basal lamina (for details, see below and Figure 1). Indeed, it was previously shown that inhibition of αvβ3 integrin impairs the proliferation and self-renewal ability of bRG in ferrets (Fietz et al., 2010). On the contrary, the activation αvβ3 integrin promotes proliferative divisions of bIPs in mice by increasing the rate of cell cycle re-entry (Stenzel et al., 2014). Interestingly, mouse bIPs are dependent on the availability of thyroid hormones to undergo proliferative divisions possibly due to the lack of endogenous ECM production in the developing cortex. A study focused on the molecular identity of human bRG showed that bRG produce local ECM components that are likely needed to sustain their proliferation in the SVZ (Pollen et al., 2015). For example, tenascin (TNC), an extracellular glycoprotein highly expressed in bRG, supports stem cell maturation by regulating the local concentration of fibroblast growth factors (Garwood et al., 2004; Barros et al., 2011). These findings highlight that increased cell-autonomous ECM production is among the critical factors underlying the enhanced proliferative and self-renewal capacity of basal progenitors in the SVZ. Overall, interactions with ECM components are among the key factors that shape the stem cell niche in the SVZ (Figure 1). ECM contributes to this niche using two strategies: i) through interactions between the basal process and the basal lamina at the pial surface (for most bRG), and ii) by local deposition of ECM components in the SVZ (for bRG and bIPs).

However, ECM alone would not be sufficient to sustain a proliferative niche in the SVZ. Proliferation is a metabolically demanding process that requires sustained delivery of oxygen and nutrients to the proliferating basal progenitors (Palmer et al., 2000; Shen et al., 2004; Vogenstahl et al., 2022), necessitating the involvement of another system, i.e., the vascular system.

## 3 The role of vasculature in establishing the SVZ stem cell niche

Vasculature is an essential component of the developing and adult mammalian brain. It does not only serve as a major supply of nutrients, oxygen and signaling factors for all neural cells but also plays a role in the regulation of the proliferation of neural stem cells during fetal and adult neurogenesis (Palmer et al., 2000; Segarra et al., 2019; Peguera et al., 2021). In adult SVZ, the neural stem and progenitor cells are located nearby blood vessels (Shen et al., 2008). The contact between these cells and blood vessels are generally lack astrocytes and pericytes, suggesting that signaling factors from blood vessels can directly access adult neural stem and progenitor cells (Kojima et al., 2010). While these data indicate the importance of the stem cell niche and notably of vasculature in the adult SVZ, the focus of this review is the SVZ stem cell niche during fetal neocortex development. For more information about the adult SVZ niche, we would like to refer to the following excellent reviews about this topic (Alvarez-Buylla et al., 2002; Ming and Song, 2011; Gonçalves et al., 2016; Jurkowski et al., 2020). During fetal development, there is a high demand for the above-mentioned supplies by neural stem and progenitor cells (Kashiwagi et al., 2023). Communication between the nervous and vascular systems is critical to ensure the proper development and function of the brain (Peguera et al., 2021; Vogenstahl et al., 2022). The first interaction between these systems emerges at around embryonic day (E) 8.5 in the mouse with the appearance of a vascular mesh surrounding the neural tube–the so-called perineural vascular plexus (PNVP) (Peguera et al., 2021). This is followed by the branching of newly developed vessels and the formation of intraneural vascular plexus at around E10.5, which coincides with the onset of neurogenesis. The expanding brain vasculature extends into the ventricles where it forms new branches surrounding the ventricle, and then migrates in reverse direction reaching to the pial surface (James et al., 2011; Segarra et al., 2019). In the developing cortex, the blood vessels establish a close relationship with neural stem and progenitor cells (Tata et al., 2015; Tata and Ruhrberg, 2018; Karakatsani et al., 2019; Komabayashi-Suzuki et al., 2019). The vascular sprouts from the periventricular plexus grow close to the soma of aRG on the ventricular surface, while basal processes of aRG and bRG lay perpendicular to the PNVP at the pial surface (Paredes et al., 2018; Segarra et al., 2019). Recently, it has become clear that blood vessels not only deliver nutrients and oxygen but are also involved in the regulation of neurogenesis (Karakatsani et al., 2019; Vogenstahl et al., 2022). Co-culture of neural stem cells (NSCs) and endothelial cells has shown that endothelial cells secrete soluble factors that promote the proliferation of NSCs, and the absence of endothelial cells results in the differentiation of NSCs (Shen et al., 2004)(Figure 1). Consistent with this finding, devascularization and hypoxia induced by the deletion of neural VEGF-A decreases progenitor cell proliferation in the VZ and SVZ of developing mouse telencephalon (Haigh et al., 2003). These studies highlight the important role of blood vessels in maintaining the stem cell niche in the germinal zones of the mammalian cortex.

Blood vessels in the brain include endothelial cells, which form a layer lining the blood vessels and regulate the exchange of nutrients and oxygen between blood stream and brain tissue. Endothelial cells start emerging in the VZ and SVZ of the mouse cortex by E13 and generate a honeycomb-patterned vascular plexus within the SVZ (Vasudevan et al., 2008; Javaherian and Kriegstein, 2009). The potential role of brain vasculature in providing a stem cell niche in the SVZ has emerged from studies of mouse bIPs (Javaherian and Kriegstein, 2009; Stubbs et al., 2009). These studies showed that proliferating bIPs follow the temporal and spatial pattern of developing vasculature in the SVZ of mouse embryonic cortex. Importantly, these bIPs undergo mitosis near the branch points of vasculature and align adjacent to newly forming blood vessels, relating cortical neurogenesis to angiogenesis in the developing cortex (Javaherian and Kriegstein, 2009). As mentioned earlier, bIPs lack both apical and basal processes, which can receive signals from the apical and/or the basal side of the cortical wall, therefore these cells rely on the local microenvironment to sustain their proliferation (Javaherian and Kriegstein, 2009; Arai et al., 2011). This suggests that endothelial cells from the brain vasculature provide a stem cell niche for the proliferation of bIPs within the SVZ. Additionally, a later study has confirmed the physical interaction between progenitors and filopodia of the endothelial tip cells, which is a hallmark cell type for active angiogenesis (Di Marco et al., 2020). This finding further showed that blood vessels could contribute to the proliferative niche not only through secreted factors but also via direct cell-cell contacts. In this context, it is interesting to highlight that blood vessels also possess a basal lamina, which could contribute to the stem cell niche in the SVZ by providing proliferative signals using cell-cell contacts. This could be particularly important for proliferative bIPs, and bRG lacking basal process, which do not have contact with the basal lamina at the pial surface due to the lack of a basal process. However, the exact mechanism by which the vascular system provides such a proliferative niche in the SVZ remains to be explored. The major open questions to investigate include: What is the exact repertoire of cues secreted from vasculature to support proliferation in SVZ? Are there physical interactions between endothelial cells and, bRG, e. g., between bRG processes and endothelial tip cells, which could regulate the proliferative capacity of these cells in the developing cortex (see Figure 1)?

## 4 Diverse BP morphology–an adaptation to the SVZ stem cell niche

After delaminating from the ventricular surface, BPs migrate and establish a stem cell niche in the SVZ. Lacking the apical contact, hence the access to the pro-proliferative signals from the ventricle, BPs crucially depend on signaling molecules from different sources in the developing cortex (as discussed above). Therefore, they need to adopt new strategies to maximize their ability to sense pro-proliferative signals to sustain their proliferative capacity.

An important cell biological factor underlying the high proliferative capacity of bRG emerges from their dynamic and diverse morphologies (Figure 1). Initially, bRG were defined as monopolar cells that have only a basal process extending to the basal lamina at the pial surface (Fietz et al., 2010; Hansen et al., 2010; LaMonica et al., 2012). Later, new bRG subtypes exhibiting diverse morphologies have been identified in ferret and macaque developing neocortex (Betizeau et al., 2013; Kalebic et al., 2019), revealing the complexity and heterogeneity of bRG morphology. Betizeau and colleagues showed that bRG can be found in multiple morphotypes in the developing macaque cortex, including bRG with only a basal process (bRG-basal-P; -P for process), bRG with only an apically directed process (bRG-apical-P), and bRG with an apically directed process and a basal process (bRG-both-P) (Betizeau et al., 2013). In a later study, Kalebic and colleagues described two additional bRG morphotypes found in human and ferret, but not in mouse neocortex: bRG with two basal processes, and bRG with an apically directed and two basal processes (Kalebic et al., 2019). Moreover, increasing proliferative capacity of bRG was found to be correlated with the increasing number of bRG processes, highlighting a key role of bRG morphology for their high proliferative capacity (Betizeau et al., 2013; Kalebic et al., 2019; Kalebic and Huttner, 2020).

The long basal process of bRG has been implicated in the proliferation of bRG by receiving and transmitting the extrinsic proliferative signals from the basal lamina (Penisson et al., 2019; Ferent et al., 2020b). The signaling molecules and the growth factors in the basal lamina can be detected at the tip of the basal process called the basal endfoot, which provides an essential platform for communication between ECM in the basal lamina and the cell body (Bjornsson et al., 2015). In addition to basal processes, another interesting morphological feature of bRG is small protrusions called lamellate expansions. These short processes are found in aRG and bRG of gyrencephalic species and their numbers increase during development (Kalebic et al., 2019). These short expansions might play a role in sensing pro-proliferative signals from the environment, including local ECM, blood vessels, neurons migrating along the basal process and axons (Kalebic and Huttner, 2020). Hence, lamellate expansions can serve as another strategy to adapt and maintain the stem cell niche in the SVZ.

In addition to bRG, bIPs also show morphological features that are linked to their proliferative capacity. As mentioned earlier, human bIPs show higher proliferative capacity than mouse bIPs (Fietz et al., 2010; Betizeau et al., 2013). Multipolar bIPs in human neocortex have larger numbers of processes than ferret and mouse bIPs (Kalebic et al., 2019). Accordingly, it has been shown that reduction in the number of bIP processes in human neocortex leads to decreased proliferation. On the other hand, expanding the number of bIP processes in mouse cortex increases their proliferative capacity by activation of integrin signaling, which is critical for BP proliferation and self-renewal as described above (Kalebic et al., 2019).

In conclusion, the dynamic behavior and the diverse morphologies displayed by BPs support their adaptation to the stem cell niche in the SVZ by mediating access to pro-proliferative signals. Therefore, it is conceivable that BP morphology is critical to establish and maintain the stem cell niche in the SVZ.

## 5 Brain organoids–a model for bRG studies

As mentioned earlier, bRG are thought to be one of the key cell types underlying neocortical expansion in human during brain evolution. As the mouse cortex shows low abundance and basically no morphological diversity in this cell type, bRG cannot be well assessed using this animal model. 2D *in vitro* and *ex vivo* cultures, such as organotypic slice cultures, have shed light on the human-specific features of bRG during neocortex development (Betizeau et al., 2013; Kalebic et al., 2019). However, these models fail to provide spatial complexity of the developing human neocortex and are not compatible with monitoring development for extended time periods. To delineate the complex and dynamic features of bRG within their specialized niche, it is essential to develop *in vitro* systems that can faithfully recapitulate dynamic processes of human neocortical development for extended periods of time.

The culture of cells in a three-dimensional (3D) setting enabled the development of *in vitro* neural models that can more accurately represent the complexity and cellular diversity of a developing brain (Hopkins et al., 2015; Chandrasekaran et al., 2017; Jalink and Caiazzo, 2021). The first 3D neural culture system, so called neurospheres, were generated from neural stem cells (Reynolds and Weiss, 1992). The neurospheres offer a simple but effective approach to investigate self-renewal capacity and multipotency of neural stem and progenitor cells (NSPCs) (Vescovi et al., 1999; Jensen and Parmar, 2006; Jalink and Caiazzo, 2021). The NSPCs in the neurospheres give rise to different types of neurons and glial cells. However, neurospheres are limited to model specific cytoarchitecture of brain tissue (Qian et al., 2019). Recently, the establishment of induced pluripotent stem cells (iPSCs) from somatic cell reprogramming has paved the way to produce many cell types of interest *in vitro* (Takahashi and Yamanaka, 2006; Takahashi et al., 2007). Moreover, the culture of iPSCs in a three-dimensional (3D) environment has led to the establishment of various pluripotent stem cell-derived *in vitro* tissue models called organoids (Sato et al., 2009; Eiraku et al., 2011). Three-dimensional (3D) *in vitro* models of brain tissue, i.e., brain organoids, have given a unique opportunity to study human-specific features of brain development. Brain organoids are 3D tissue structures generated from pluripotent stem cells (iPSCs or ESCs) that recapitulate certain key processes of brain development to achieve various cellular and structural features of the fetal brain. Since they provide an opportunity to access developing human brain tissue *in vitro*, they have served as robust models to study human-specific features of neurodevelopment and disease mechanisms (Lancaster et al., 2013; Mora-Bermúdez et al., 2016; Qian et al., 2016; Li et al., 2017; Heide et al., 2018; Kanton et al., 2019; Benito-Kwiecinski et al., 2021; Johansson et al., 2022). Subsequently, the generation of nonhuman primate brain organoids enabled the identification of key molecular and cellular mechanisms underlying the differences between developing human and non-human primate neocortex. Therefore, brain organoids have emerged as a reliable model to study key aspects and features of neocortical development, diseases, and evolution.

Given their ability to mimic key features of the developing human neocortex, cerebral organoids are promising as a suitable model system for studying bRG biology. Although cerebral organoids do not fully capture the abundance of bRG in the fetal human neocortex, they are able to provide insights into mechanisms underlying the high proliferative capacity of bRG.

Transcriptomic comparisons between human fetal brain tissue and cerebral organoids confirmed the presence of a cell population with bRG signature in brain organoids validating them as potential models for studies on bRG (Pollen et al., 2015; Bershteyn et al., 2017; Pollen et al., 2019). Brain organoids have proven to be useful for identifying inter-species differences in molecular and cellular aspects of bRG. In this context, transcriptomic studies performed in human and non-human primate brain organoids have found an increased activation of PI3K-AKT-mTOR pathway in bRG of human organoids (Pollen et al., 2019). Moreover, brain organoids can be used to study functional effects of genetic manipulations on bRG (Fischer et al., 2022; Pinson et al., 2022), as seen in a recent study identifying the role of the human-specific gene *ARHGAP11B* in the proliferative capacity of bRG. Upon expression of *ARHGAP11B*, the number of bRG increased in chimpanzee cerebral organoids. On the other hand, the knock-out of *ARHGAP11B* resulted in a decrease of the bRG pool in human brain organoids that could be restored by overexpression of *ARHGAP11B* (Fischer et al., 2022). These studies highlight that brain organoids are promising tools to investigate the role of bRG in human neocortex development and evolution.

However, brain organoids still pose certain limitations that may underlie the low abundance of bRG in this model system. As mentioned above, the microenvironment of the developing cortex provided in SVZ, particularly in the oSVZ, is critical for the maintenance of bRG proliferation and self-renewal. In this context, the following limitations of brain organoids are relevant. Firstly, the ECM in brain organoids is not precisely defined and often relies on the addition of commercially available basement membrane matrices such as Matrigel (Heo et al., 2022). It is still not well studied to what extent organoids can recapitulate the tissue-specific ECM composition of the developing neocortex. Furthermore, it remains elusive whether brain organoids could model the spatial distribution of ECM components and dynamic cell-ECM interactions of the fetal neocortex. It is important to note that many ECM components are not present in organoids due to the absence of cell types, like endothelial and meningeal cells, which produce these ECM components. Secondly, brain organoids do not have endogenous vascularization, leading to limited oxygen and nutrient diffusion to the interior parts of the organoid (Cakir et al., 2019; Qian et al., 2019; Lamontagne et al., 2022). The lack of sufficient nutrient and oxygen supply, which is highly demanded by bRG, likely hampers the survival and proliferation of these progenitors in brain organoids (Karakatsani et al., 2019; Matsui et al., 2021). In addition to that, secreted molecules from blood vessels and the interactions between bRG and basal lamina of the blood vessels are speculated to promote the proliferation of bRG (Nikolova et al., 2007; Goldberg and Hirschi, 2009). Therefore, the lack of blood vessels and tissue-specific ECM composition are among the critical limitations that need to be addressed to make brain organoids more reliable models for studying human bRG.

However, this incomplete modeling of the SVZ stem cell niche in brain organoids can also be seen as a chance to identify and study the components and functions of this niche by introducing these missing components into brain organoids.

## 6 Conclusion and perspectives

The establishment and maintenance of the stem cell niche in the SVZ is essential for proliferation and self-renewal of basal progenitors residing therein. In light of previous findings, it is conceivable to suggest that tissue-specific ECM composition and blood vessels are critical sources supporting the stem cell niche in the SVZ. To maintain their remarkable proliferative capacity within the SVZ, basal progenitors, notably bRG, develop dynamic and diverse morphologies, which may improve their ability to sense their microenvironment and to receive pro-proliferative signals. The stem cell niche that supports bRG proliferation is also relevant in the context of a highly invasive brain tumor, glioblastoma. It has been shown that glioblastoma contain a proliferative cell population that shares a similar transcriptional signature with bRG. These bRG-like cells express certain bRG markers that mediate ECM interactions, and display bRG-like morphology, which could promote the expansion and the invasiveness of glioblastoma tumors (Bhaduri et al., 2020; Wang et al., 2021). This suggests that a similar niche that supports bRG proliferation could be acquired in glioblastoma to provide a microenvironment permissive for tumor expansion. Therefore, understanding the stem cell niche in the SVZ is critical to gain further insights into the microenvironment of glioblastoma, which provides essential knowledge for the development of therapeutic strategies. However, the limitations of *in vivo* and *in vitro* models for the developing human neocortex make it difficult to investigate the dynamic microenvironment within the SVZ and its effect on the proliferative capacity of bRG. The continuous improvement of brain organoids offers a unique opportunity to address fundamental questions in neocortical development. Nonetheless, brain organoids still cannot fully recapitulate the features of fetal human neocortex, including the high abundance of bRG, suggesting that the SVZ stem cell niche is not well reproduced in this model system. In this context, future studies are needed to specify the exact ECM composition and extrinsic factors that contribute to the stem cell niche in the SVZ. Additionally, the extent of interactions between vascular cells and basal progenitors needs to be further explored to identify the repertoire of factors provided by blood vessels required for the proliferation of basal progenitors. Previous strategies to induce vascularization in brain organoids have yielded an improved organoid survival and neuronal maturation (Mansour et al., 2018; Cakir et al., 2019; Shi et al., 2020; Matsui et al., 2021). It remains to be elucidated whether vascularization improves the abundance of basal progenitors, particularly of bRG, in brain organoids. Hence, it would also be interesting to investigate whether the positioning of blood vessels plays a role in establishing bRG morphology, thereby contributing to their proliferative capacity. Future studies to incorporate ECM components and blood vessels in brain organoids are likely to advance the ability of this model system to recapitulate the unique features of bRG and the mechanisms underlying their high proliferative capacity. Future efforts in this direction will make organoids a more reliable and powerful tool to study human neocortical development and its evolution. Finally, these “improved” organoids could also be used to study neurodevelopmental diseases, which are caused by abnormal ECM formation and/or disturbed vascularization (e.g., DiGeorge’s syndrome, autism spectrum disorder (ASD) (Chakravarti et al., 2022; Ishihara et al., 2023). In such cases, these brain organoids can be genetically modified to generate specific disease model and be used to study disease mechanisms (Fischer et al., 2019). Moreover, brain organoids generated from patient-derived iPSCs are not only useful to investigate disease mechanisms but also provide an unprecedented opportunity to explore strategies for personalized therapeutics.
